# Sex-Related Differences in Clinical Features, Neuroimaging, and Long-Term Prognosis After Transient Ischemic Attack

**DOI:** 10.1161/STROKEAHA.120.032814

**Published:** 2021-01-26

**Authors:** Francisco Purroy, Mikel Vicente-Pascual, Gloria Arque, Mariona Baraldes-Rovira, Robert Begue, Yhovany Gallego, M. Isabel Gil, M. Pilar Gil-Villar, Gerard Mauri, Alejandro Quilez, Jordi Sanahuja, Daniel Vazquez-Justes

**Affiliations:** Stroke Unit, Department of Neurology, Hospital Universitari Arnau de Vilanova de Lleida, Spain (F.P., M.V.-P., M.B.-R., Y.G., M.P.G.-V., G.M., A.Q., J.S., D.V.-J.). Clinical Neurosciences Group, Institut de Recerca Biomèdica de Lleida (IRBLleida), Universitat de Lleida, Spain (F.P., M.V.-P., G.A., R.B., M.I.G., M.P.G.-V., G.M., A.Q., J.S., D.V.-J.).

**Keywords:** atherosclerosis, incidence, ischemic attack, transient, prognosis, risk factors

## Abstract

Differences in sex in the incidence, presentation, and outcome of events after ischemic stroke have been studied in depth. In contrast, only limited data are available after transient ischemic attack (TIA). We aim to assess sex-related differences in the presentation, cause, neuroimaging features, and predictors of long-term prognosis in patients with TIA.

**Methods:**

We carried out a prospective cohort study of consecutive patients with TIA from January 2006 to June 2010. Nondefinitive TIA events were defined by the presence of isolated atypical symptoms. The risk of stroke recurrence (SR) and composite of major vascular events were stratified by sex after a median follow-up time of 6.5 (interquartile range, 5.0–9.6) years.

**Results:**

Among the 723 patients studied, 302 (41.8%) were female and 79 (10.9%) suffered a nondefinitive TIA event. Vascular territory diffusion-weighted imaging patterns (odds ratio, 1.61 [95% CI, 0.94–2.77]), and nondefinitive TIA events (odds ratio, 2.66 [95% CI, 1.55–4.59]) were associated with women, whereas active smoking (odds ratio, 0.30 [95% CI, 0.15–0.58]) and large artery atherosclerosis causes (odds ratio, 0.50 [95% CI, 0.29–0.83]) were related to men. The risk of SR was similar in both sexes (12.6% [95% CI, 8.9–16.3] for women versus 14.3% [95% CI, 11.0–17.6] for men). In contrast, the risk of major vascular events was significantly lower in women than in men (17.5% [95% CI, 13.2–21.8] versus 23.8% [95% CI, 19.7–27.9]). In both sexes, after adjusting for age, large artery atherosclerosis was associated with SR (hazard ratio, 3.22 [95% CI, 1.42–7.24] and hazard ratio, 2.00 [95% CI, 1.14–3.51]). In a Kaplan-Meier analysis, females with positive diffusion-weighted imaging (*P*=0.014) and definitive TIA (log-rank test *P*=0.022) had a significantly higher risk of SR.

**Conclusions:**

Despite similar risks of SR, there were sex-related differences in baseline characteristics, presenting symptoms, patterns of acute ischemic lesions, cause, and outcomes. These findings encourage further research into optimal preventive strategies that take into account these differences.

Differences between sexes in the incidence, presentation, and outcomes of ischemic stroke have already been studied in depth. Compared with men, women exhibit a lower incidence of stroke^1^ but a higher prevalence of important vascular risk factors for stroke, such as atrial fibrillation.^2,3^ Women are more likely to be older when they suffer strokes and have more severe strokes and poorer outcomes.^2,3^ Even so, only limited data are available about differences in sex relating to its clinical presentation, cause, imaging features, and prognosis after a transient ischemic attack (TIA). Yu et al^4^ recently observed a similar risk of subsequent vascular events between sexes in a prospective cohort of patients with minor stroke. The early risk of stroke after a TIA is high^5,6^ and urgent management is required^7^; however, few studies have focused on the long-term risk of stroke after TIA.^8,9^

Our aim was to assess differences between sexes in baseline characteristics, presentation, cause, neuroimaging features, and predictors of long-term prognosis of consecutive patients with TIA attended at an emergency department.

## Methods

### Data Availability Statement

Requests for access to the data reported in this article will be considered by the corresponding author.

### Design and Study Population

We carried out a registry-based cohort study, from January 2006 to June 2010, which included consecutive patients with TIA who had been attended by a stroke neurologist working in the emergency department of a university hospital during the first 24 hours after the onset of symptoms (Registro de pacientes con AIT de Lleida [REGITELL] registry).^10^ We did this following the Strengthening the Reporting of Observational Studies in Epidemiology statement.^11^ TIA was defined as a reversible episode of neurological deficit of ischemic origin that was fully resolved within 24 hours.^12^ Multiple TIAs were defined as the occurrence of at least 2 TIAs—the index TIA and one other TIA—in the 7 days before the index event.^13^ The typologies of the clinical symptoms were recorded using a standard form. The duration of symptoms was verified on admission and on day 7 of the follow-up period. Their respective ABCD2 risk scores (age, blood pressure, clinical features, symptom duration, and diabetes mellitus)^14^ were determined prospectively. Any potential, yet not definitive, TIA events were defined by the presence of isolated, atypical symptoms such as unsteadiness; diplopia; dysarthria; partial sensory deficit; and unusual cortical vision.^15^ All the cases were reviewed by the senior neurologist (Dr Purroy) and classified prospectively. Patients were classified as having suffered either a definitive or a nondefinitive TIA event before magnetic resonance imaging (MRI) had been performed.

Patients underwent ECG, blood tests, computed tomography brain imaging, and extracranial and intracranial ultrasound imaging as first-line investigations.^10^ In addition, those without contraindications were evaluated by cranial MRI within 7 days of symptom onset. Before the MRI examination, all the cases were studied using a nonenhanced computed tomography. Data from patients who suffered early stroke recurrence (SR) before having an MRI examination were excluded from the study. The cranial MRI included diffusion-weighted imaging (DWI) sequences. Two neuroradiologists, who were blinded to clinical features, established the presence of DWI abnormalities.^10^ Patterns of acute ischemic lesions were also recorded. The lesion patterns identified by the DWI were classified into single lesions (cortical in one vascular territory, or subcortical); scattered lesions (numerous lesions) in one vascular territory; and multiple lesions in multiple vascular territories.^10^ The interobserver agreement (kappa value) was 1.0 for positive DWI and 0.98 for DWI patterns.

### Outcomes and Follow-Up

The primary outcome was the occurrence of recurrent ischemic stroke. This was defined as the appearance of new focal symptoms or signs associated with acute ischemic changes shown on neuroimaging, brain computed tomography, or MRI. Recurrent TIAs, which were defined as new neurological symptoms or a deficit lasting for <24 hours with no new infarction evident in neuroimaging, were not included in the primary outcome. The secondary outcome was a composite of major vascular events (MVE) that included acute coronary syndrome, which were defined as myocardial infarction—either with or without an increase in ST-segment–elevation—or as unstable angina followed by urgent catheterization, recurrent ischemic stroke, the development of symptomatic peripheral arterial disease, and death from cardiovascular causes. Deaths from cardiovascular causes included fatal acute coronary syndrome, fatal stroke, fatal intracranial hemorrhage, fatal pulmonary embolism, sudden death, and unobserved or unexpected death within 30 days.^8^ The risk for each individual component of MVE was determined for each sex. Structured clinical visits were performed by a stroke physician (Dr Purroy, Baraldes-Rovira, Gallego, Gil-Villar, Mauri, Quilez, Sanahuja, Vazquez-Justes, Vicente-Pascual) during the follow-up period. They were made at 7 days, 3 months, 1 year, 5 years, and 10 years. If a patient moved out of the local area or travel to the hospital was impossible, the follow-up was conducted by phone. Recurrent events and outcomes were also actively identified by an annual review of electronic medical records.

### Classification of Stroke Subtypes

Patients were classified etiologically based on the ORG 10172 Trial (TOAST [Trial of ORG 10172 in Acute Stroke Treatment])^16^ definitions. The causes identified were large artery occlusive disease (LAA), small vessel disease, cardioembolic, uncommon, or undetermined causes. The TOAST classification was performed at discharge, once the diagnostic workup was complete. In cases in which the initial classification was undetermined, the subsequent appearance of a cardioembolic source, during the follow-up, changed the stroke subtype classification. Patients were classified as having LAA if they were found to exhibit a symptomatic, moderate to severe, intracranial or extracranial, stenosis.^10^ Patients exhibiting what were suspected of being paradoxical emboli resulting from patent foramen ovale, in the absence of LAA, were classified as cardioembolic.

### Statistical Analysis

We compared the baseline characteristics, cause, presence, and distribution of acute lesions in DWI, ABCD2 score,^14^ and outcomes between sexes. The quantitative variables were compared using either the Student *t* test or the Mann-Whitney *U* test. The qualitative variables were compared using the χ^2^ test or Fisher exact test when the expected cell frequency was <5. A Bonferroni correction (a multiple-comparison correction) was applied to all the significant associations to reduce the risk of finding false-positive associations.

We used multivariable logistic backward stepwise regression analysis with an adjustment for clinical variables (model 1) and neuroimaging features (model 2) to obtain adjusted odds ratios. Variables for which *P*<0.10 in univariate testing were included. The cumulative risks of recurrent stroke and MVE during follow-up were estimated using Kaplan-Meier analysis; the results were censored at the time of the outcome event, patient death, or the end of the follow-up period. Risks were compared using the log-rank test. Data on patients with no information at 10 years were censored at the time of the last available follow-up. In addition, we performed a univariate analysis for each sex to assess variables associated with SR. Finally, a Cox proportional hazards multivariable analysis was performed with adjustments for age (model 1) and for age and neuroimaging features (model 2) to identify predictors of SR. It was also adjusted for the patient characteristics that significantly predicted outcomes in univariate logistic regression models. We compared the risk of SR in subgroups of patients categorized according to the main identified predictors, using Kaplan-Meier analysis and the log-rank test. All the tests were 2-sided. Missing data was included as a random effect when fitting the model for multivariable analyses. The statistical analysis of the data was carried out using the SPSS statistical package, version 20.0 (SPSS, Chicago, IL).

### Standard Protocol Approvals, Registrations, and Patient Consents

Written informed consent or assent from relatives was obtained for all the participants. The study was approved by our local ethics committee: the Comité d’Etica i Investigació Clínica de l’Hospital Universitari Arnau de Vilanova de Lleida.

## Results

A total of 771 patients were referred as possible TIA. Forty-eight patients were diagnosed as mimics and were excluded, leaving 723 for analysis. Three hundred and two of the patients studied (41.8%) were female. The median (percentiles 5% and 95%) follow-up time was 6.5 (5.0-9.6) years. Seven hundred twenty of the 723 (99.6%) patients studied were admitted to the Neurology department. Of the 614 (84.9%) patients who underwent DWI (4.0 [SD 1.8] days after the index event), acute ischemic lesions were identified in 244 (39.7%). Two hundred and thirty-three (32.2%) patients died during the follow-up period. Ninety-eight patients (13.6%) suffered SR. There were 57 (58.1%) events in the first year, 26 (26.5%) between the second and fifth years, and 15 (15.3%) between the sixth and tenth years. MVE was observed in 153 (21.2%) patients. Eighty-two (53.6%) cases of MVE occurred in the first year, 45 (29.4%) between the second and fifth years, and 26 (17.0%) between the sixth and tenth years.

### Variables Associated With Sex

As shown in Table 1, there were sex-associated differences in the baseline characteristics, clinical presentation, cause, and neuroimaging features. The women were older than the men (72.4 [SD: 11.3] versus 69.5 [SD: 12.3] years; *P*=0.001). Fewer of the women were active smokers (15 [5.0%] versus 87 [20.75%]; *P*<0.001) or had a history of alcoholism (0 versus 21 [5.0%]; *P*<0.001) or peripheral arterial disease (5 [1.7%] versus 21 [5.0]; *P*=0.024). In addition, hypertension tended to be more frequent in the women than in the men (213 [70.5%] versus 268 [63.7%]; *P*=0.053) and ischemic heart disease was less frequent in the women than in the men (33 [10.9%] versus 66 [15.7%]; *P*=0.067). We also observed a lower proportion of multiple TIA events (58 [19.2%] versus 107 [25.4%]; *P*=0.050) and higher proportion of nondefinitive TIA events in the women than in the men. The women tended to have higher levels of systolic arterial pressure than the men (155.0 [SD, 30.2] versus 151.2 [SD, 26.7] mm Hg; *P*=0.081) and to suffer a higher proportion of speech impairment events (199 [65.9%] versus 250 [59.4%]; *P*=0.075). LAA was overrepresented in men with respect women (89 [21.1%] versus 36 [11.9%]; *P*=0.001). Although no significant differences were observed between sexes regarding the proportion of positive DWI, it was more frequent to find scattered lesions in one vascular territory pattern in the men than in the women (66 [44.0%] versus 26 [27.4%]; *P*=0.009).

**Table 1.**
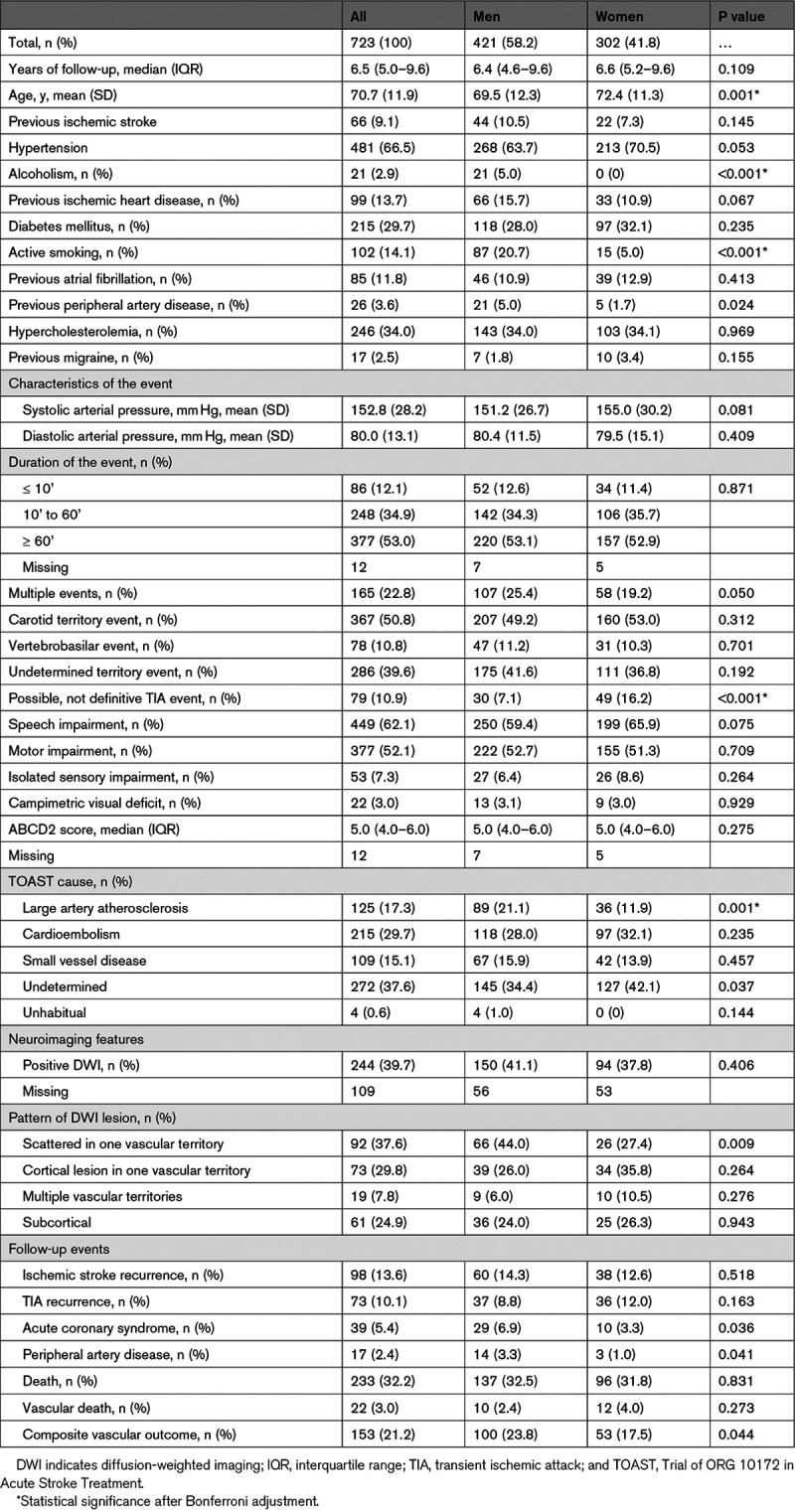
Clinical Characteristics, Neuroimaging Features, and Outcomes by Sex

Table 2 shows the multivariable logistic regression analysis. Nondefinitive TIA events were associated with women (odds ratio, 2.45 [95% CI, 1.48–4.04]; *P*<0.001), whereas active smoking (odds ratio, 0.24 [95% CI, 0.13–0.43]; *P*<0.001), LAA (odds ratio, 0.54 [95% CI, 0.35–0.83]; *P*=0.005), and scattered lesions in one vascular territory pattern (odds ratio, 0.22 [95% CI, 0.04–0.108]; *P*=0.061) were related to men.

**Table 2.**
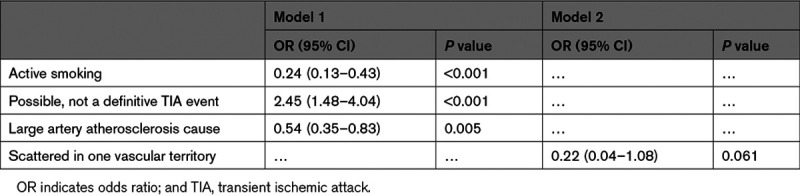
Logistic Regression Analysis of Variables Associated With Sex Female

### Outcome Events by Sex

**Figure 1.**
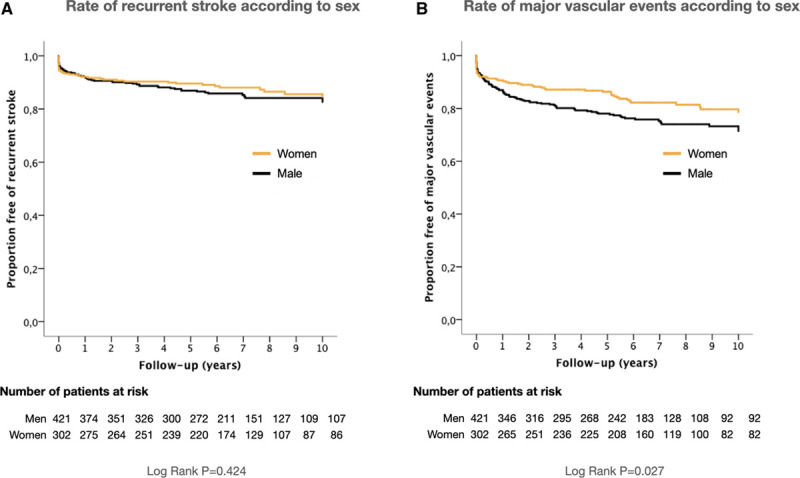
**Kaplan-Meier event curves for stroke recurrence and major vascular events according to sex.** **A**, Rate of recurrent stroke according to sex; (**B**) rate of major vascular events according to sex.

As shown in Table 1 and Figure 1, the risk of SR after the end of the follow-up was similar for both sexes (12.6% [95% CI, 8.9–16.3] for women versus 14.3% [95% CI, 11.0–17.6] for men; *P*=0.518; log-rank test *P*=0.424). Similarly, no significant differences between sexes were observed in the risk of TIA recurrence (12.0% [95% CI, 8.3–15.7] versus 8.8% [95% CI, 6.1–11.5; *P*=0.163). In contrast, the risk of MVE was significant lower in women than in men (17,5% [95% CI, 13.2–21.8] versus 23.8% [95% CI, 19.7–27.9]; *P*=0.044; log-rank test *P*=0.027). A similar situation was observed with regard to the risk of suffering acute coronary syndrome (3.3% [95% CI, 1.3–5.3] versus 6.9% [95% CI, 4.5–9.3]; *P*=0.036) or peripheral arterial disease (1.0% [95% CI, 0–2.1] versus 3.3% [95% CI, 1.6–5.0]; *P*=0.041). No significant differences between sexes were observed in the risk of death (31.8% [95% CI, 26.5–37.1] versus 32.5% [95% CI, 28.0–37.0; *P*=0.163).

### Predictors of Outcomes by Sex

**Figure 2.**
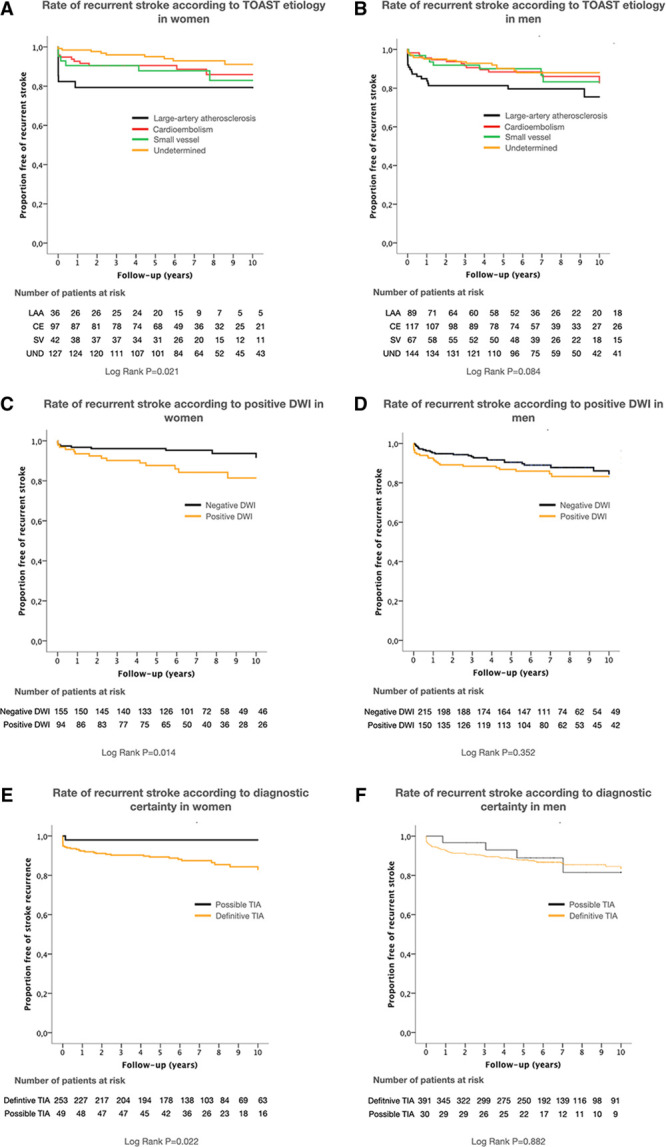
**Kaplan-Meier event curves.** Kaplan-Meier event curves for stroke recurrence according to TOAST (Trial of ORG 10172 in Acute Stroke Treatment) cause, diffusion-weighted imaging (DWI) positivity, and diagnostic certainty in women (**A**, **C**, and **E**) and in men (**B**, **D**, and **F**). CE indicates cardioembolism cause; LAA, large artery atherosclerosis; SV, small vessel cause; TIA, transient ischemic attack; and UND, undetermined cause.

In both sexes (Tables 3 and 4), LAA was associated with SR (women: hazard ratio, 2.67 [95% CI, 1.21–5.92]; *P*=0.015 and men: hazard ratio, 2.06 [95% CI, 1.17–3.62]; *P*=0.017). As shown in Figure 2, an undetermined cause was associated with the lowest risk of SR in women. In contrast, no differences were observed among non-LAA causes in men. In women, having suffered a nondefinitive event tended to imply a lower risk of SR (hazard ratio, 0.15 [95% CI, 0.02–1.08]; *P*=0.059), whereas positive DWI was significantly related to a higher risk of SR (hazard ratio, 2.74 [95% CI, 1.19–6.33]; *P*=0.018). In the Kaplan-Meier analysis (Figure 2), females with positive DWI (log-rank test *P*=0.014) and definitive TIA (log-rank test *P*=0.022) had a significantly higher risk of SR throughout follow-up; in contrast, men with positive DWI had a significantly higher risk of SR just during the first 2 years of follow-up (log-rank test *P*=0.044).

**Table 3.**
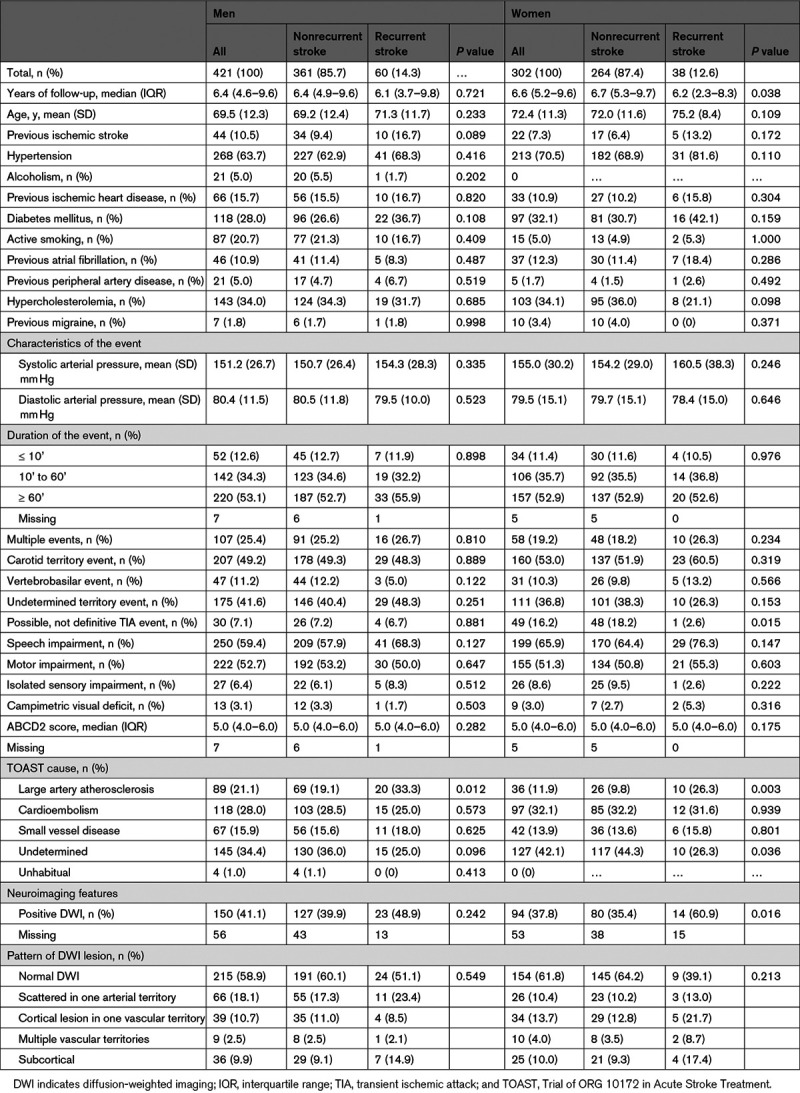
Univariate Analysis of Variables Associated With Stroke Recurrence by Sex

**Table 4.**
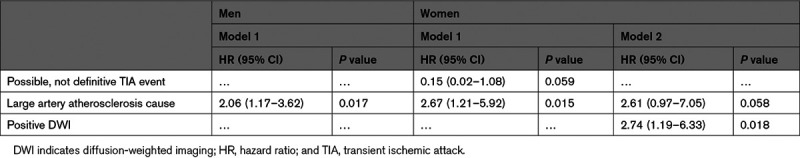
Cox Proportional Hazards Regression Model to Assess Risk of Subsequent Stroke After TIA, by Sex

## Discussion

In our study of high-risk patients with TIA attended to at an emergency department, we found differences between sexes in baseline and clinical characteristics, cause, neuroimaging features, long-term outcomes, and predictors of SR. We observed that women were older, had a higher proportion of possible TIA events, and a larger number of events of undetermined cause than men. In contrast, men accumulated more modifiable vascular risk factors, such as smoking, and had a higher proportion of LAA events than women. Interestingly, although no differences were observed in the risk of SR, positive DWI was only a predictor of SR in women. Having suffered a possible TIA event was associated with a low risk of SR in women. LAA emerged as a predictor of SR in both sexes, while men had a higher risk of MVE than women. Some of the findings were consistent with previous reports on stroke patients, whereas others revealed specific features of patients with TIA. Previous stroke studies observed significant differences in age between sexes.^2,3,17,18^ This situation could be explained by the longer life expectancy of women in our country.^19^ Differences in modifiable vascular risk factors had been described previously^2,3,17,18,20,21^ and explain the sex-difference in the risk of MVE.^9,22,23^ Prior stroke studies^2,17,21^ reported an overrepresentation of LAA in men, as they accumulate more vascular risk factors. The higher proportion of undetermined cause events in women than in men and the differences in DWI pattern lesions highlighted the differences in stroke cause between sexes and the need to improve diagnostic work in women to reduce the proportion of embolic strokes of undetermined sources. In previous stroke studies, being female was clearly associated with atrial fibrillation.^2,3,18,20^ However, limited data exist on atrial fibrillation detection after TIA. Furthermore, management settings and diagnostic pathways frequently differ substantially between stroke and patients with TIA.^24^ Most management strategies applied to patients with TIA have been established to prevent early SR in LAA patients.^25^

In a recent study involving 1648 patients suspected of having suffered a minor stroke event, Yu et al^4^ observed that despite men and women presenting similar symptoms, the latter were more likely to receive a diagnosis of stroke mimic. In our study, nondefinitive TIA was more frequent among women than men. The proposed explanations for the observed differences between sexes include more nonischemic causes of transient neurological attacks, such as migraine^3,26^ and more atypical clinical manifestations in women than in men.^27^ However, in our study the proportion of migraine was similar in both sexes, the rate of atypical TIA was lower than previously described,^15^ and no significant differences between sexes were observed for late SR. To the best of our knowledge, this is the first time that differences between sexes have been analyzed in a cohort of consecutive patients with TIA in long-term follow-up. Previous studies of differences between sexes in outcomes after TIA or minor strokes reported the same risk of SR after 30 days,^28^ 90 days,^4^ and 1 year^28^ of follow-up. In our study, LAA was the main predictor of SR in both sexes, with the risk of SR falling after the first five years of follow-up. Differences in the early risk of SR and after 1 year based on etiologic subtypes have already been described in depth.^29–31^ The accumulation of recurrent events during the first year of follow-up, affecting slightly >1 out of every 2 patients, could explain the relevance of LAA as a predictor of SR in both sexes. We observed with interest how positive DWI was associated with a high risk of SR throughout follow-up in women, but only during the first years of follow-up in men. The use of DWI is recommended not only for the tissue-based definition of TIA but also because the presence of acute DWI lesion is a well-documented predictor of 90-day and 1-year SR following a TIA.^10,30,32,33^ Only one previous study, which included 633 patients with TIA, had previously demonstrated the predictive value of DWI positivity in the 10-year risk of SR after an index TIA.^34^ In contrast, in a registry of 4789 patients with a TIA or who had suffered a minor ischemic stroke, from 21 countries, Amarenco et al^30^ reported that positive DWI was predictive of SR at 1 year but not at between 2 and 5 years.^8^ These results would seem to suggest that late SR is not directly related to the ischemic mechanism of index event.^35^ The higher proportion of nondefinitive cases of TIA found in women in our study was associated with a low risk of SR. This could also explain the significant role of DWI positivity, as it excludes the possibility of nonischemic mechanisms being responsible for transient symptoms. Moreover, differences in etiological subtypes between sexes could explain this observation. Positive DWI seemed to be a relevant predictor among early recurrences related to LAA, whereas DWI results were more relevant in patients with undetermined etiological subtypes during the whole follow-up.

The main limitation of our study was the sample size. The inclusion of more patients would give us the opportunity to improve the analysis of differences between subgroups. Furthermore, the rates of outcomes were lower than in previous studies.^9^ Our study, therefore, probably lacked the statistical power to detect other predictors. We must also acknowledge that our cohort should probably be considered a high-risk one, given that we only included patients who were attended to in the emergency room and that the median ABCD2 score was higher than in previous large studies.^7,8^ There was therefore a low to mild risk that patients with TIA could have been underrepresented.

## Conclusions

Despite similar risks of SR, there were sex-related differences in baseline characteristics, the presentation of symptoms, patterns of acute ischemic lesions, and predictors of outcome. Men also had a significantly higher risk of suffering MVE than women. These findings should encourage us to increase our efforts to improve the etiological diagnosis to reduce the rate of embolic stroke of undetermined source among women. More research is also needed to find optimal preventive strategies that take into account differences between sexes.

## Sources of Funding

This study was supported by the Catalan Autonomous Government’s Agència de Gestió d’Ajuts Universitaris i de Recerca (2017 suport a les activitats dels grups de recerca 1628) and the Instituto de Salud Carlos III (08/1398, 11/02033 and 14/01574) and the INVICTUS plus Research Network.

## Disclosures

None.

## Supplementary Material


