# Supplementary figures and images for: Sex-Related Differences in Clinical Features, Neuroimaging, and Long-Term Prognosis After Transient Ischemic Attack
Source: Stroke. 2021 Jan 26;52(2):424–33. doi: 10.1161/STROKEAHA.120.032814 (PMC7834662; doi:10.1161/STROKEAHA.120.032814)

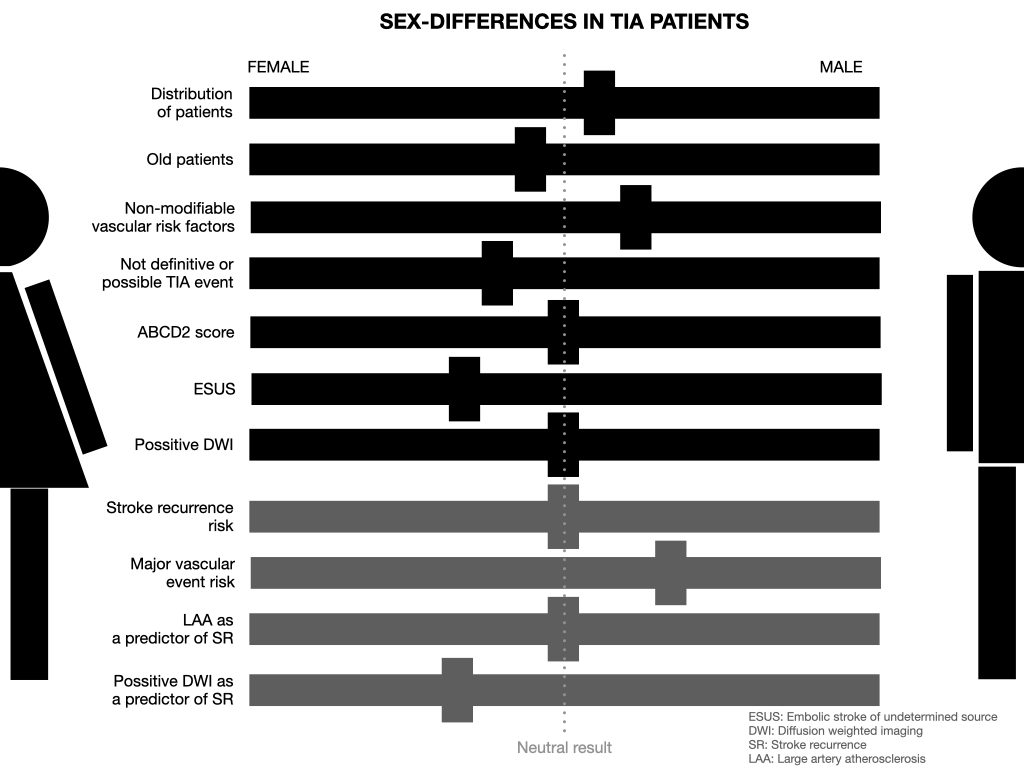

Supplement: Supplementary file 1 [file str-52-424-s001.jpg]
